# Comparative Analysis of Existing RC Columns Jacketed with CFRP or FRCC

**DOI:** 10.3390/polym10040361

**Published:** 2018-03-24

**Authors:** Marta Del Zoppo, Marco Di Ludovico, Alberto Balsamo, Andrea Prota

**Affiliations:** 1 Department of Engineering, University of Naples Parthenope, 80143 Naples, Italy; 2 Department of Structures for Engineering and Architecture, University of Naples Federico II, 80125 Naples, Italy; diludovi@unina.it (M.D.L.); abalsam@unina.it (A.B.); aprota@unina.it (A.P.)

**Keywords:** existing RC columns, CFRP, FRCC, repair, strengthening, seismic retrofit

## Abstract

Reinforced concrete (RC) columns typical of existing structures often exhibit premature failures during seismic events (i.e., longitudinal bars buckling and shear interaction mechanisms) due to the poor quality concrete and the absence of proper seismic details in the potential plastic hinge region. The Fiber Reinforced Polymers (FRP) externally bonded reinforcement is known to be a valid technique to improve the shear capacity or the ductility of existing RC columns. However, few experimental tests have proven its effectiveness in the case of columns affected by shear interaction mechanisms. In this work, the behavior of existing RC columns with border line behavior between flexure and shear have been investigated in the case of poor quality concrete and light FRP strengthening with local jacketing and medium quality concrete and strong FRP strengthening with local jacketing, in order to highlight the effect of concrete strength on the effectiveness of the retrofit intervention. As an alternative to FRP jacketing; the effectiveness of the Fiber Reinforced Cementitious Composite (FRCC) jacketing for the seismic strengthening of columns with highly deteriorated concrete cover or columns already damaged by an earthquake is also evaluated. Six full-scale RC columns have been tested under cyclic loading: one was used as a control specimen; four were strengthened in the potential plastic hinge region with carbon FRP (CFRP); and one was fully jacketed with FRCC. The comparison between poor and medium quality concrete columns showed that the CFRP local jacketing is more effective in the case of poor quality concrete. The FRCC jacketing appears to be a sound repair strategy and a suitable alternative to the FRP jacketing in case of poor quality; however, more experimental research is needed for improving this retrofit technique.

## 1. Introduction

Post-earthquakes reconnaissance [1,2,3,4] and experimental research [5,6,7,8,9,10,11,12], among many others have evidenced that existing reinforced concrete (RC) columns, designed according to old standards and construction practice, often show premature failures due to many phenomena (i.e., buckling of longitudinal bars, loss of bond, shear interaction, etc.). In particular, the cyclic loading caused by earthquakes weaken the concrete shear resisting mechanism in the plastic hinge region of existing RC members, characterized by poor quality concrete and large spacing of transverse reinforcement, also leading to brittle shear failures after the flexural yielding [13,14]. This borderline behavior of existing RC columns between flexure and shear is difficult to predict, due to the poor accuracy of assessment models [15] and can lead to premature and unpredictable structural collapses. 

To prevent the shear interaction mechanism and improve the performance of existing RC members, the use of two strengthening techniques has been herein investigated and compared: the partial jacketing of the plastic hinge region with Carbon Fiber Reinforced Polymers (CFRP) and the full-length jacketing with Fiber Reinforced Cementitious Composite (FRCC).

It is well known that the seismic performance of RC members can be improved significantly by providing an FRP external jacketing, especially in the potential plastic hinge region. A large number of experimental tests conducted on FRP jacketed RC columns under cyclic actions can be found in the literature [16,17,18], but most of these are mainly focused on the retrofitting of columns with lap spliced bars or affected by buckling phenomena. To the knowledge of the authors, very few studies have been carried out on the FRP jacketing of existing RC columns affected by shear interaction mechanisms [19].

However, in the case of poor quality concrete columns with heavily deteriorated concrete cover or columns already damaged by a seismic event, the original cover should be totally removed and replaced with a new one before the application of the FRP jacketing. Thus, an alternative strengthening technique could be the replacement of the original cover with a Fiber Reinforced Cementitious Composite (FRCC) jacket. This strengthening solution reduces time and costs of intervention if compared with the externally bonded FRP technique [20]. However, few experimental studies have been performed on RC columns jacketed with FRCC [21,22,23] to validate the effectiveness of this technique, especially in the case of columns affected by shear interaction mechanisms.

In the present work, an experimental program on six full-scale RC columns representative of existing structures and strengthened with two different jacketing techniques (CFRP and FRCC) is presented.

In the first part of the work, the effectiveness of CFRP jacketing in the potential plastic hinge region of existing RC columns, which exhibit a borderline behavior between flexure and shear, is investigated on four columns. The effect of different concrete compressive strength (poor and medium quality) and FRP reinforcement ratio on the jacketing effectiveness have been investigated. A detailed analysis of the strain distribution along the external jacketing is also reported. 

The last part of the present work investigates the use of the FRCC jacketing for the repair/strengthening of poor quality concrete RC columns already damaged by a seismic event, as an alternative to the externally bonded FRP technique. One column with poor quality concrete was pre-damaged and then repaired/strengthened with FRCC jacketing. The performance of jacketed specimens was compared in terms of failure mode, lateral capacity and ductility, stiffness degradation, energy dissipation and damage evolution.

## 2. Experimental Study

### 2.1. Test Matrix

Full-scale experimental tests on six square RC columns were performed under constant compressive axial load and cyclic lateral loading. The columns were designed accordingly to past Italian codes’ minimum requirements and old construction practice, in order to be representative of real existing structures. No longitudinal reinforcement along the cross-section secondary direction have been included, according to what was found in heavily damaged columns after the L’Aquila earthquake (2009) [4]. Transverse reinforcement details were non-conforming to current standards ACI 318 [24]: transverse reinforcement spacing *s* > *d*/3, where *d* is the cross-section effective depth, and shear contribution provided by the reinforcement (*V_s_*) lower than 3/4 of the total shear capacity. A borderline behavior between flexure and shear was expected at failure, according to refined shear capacity models [13,14]. 

One column, characterized by poor quality concrete (average compressive strength *f*_c_ = 15 MPa), was used as the control specimen to confirm the predicted failure mode. Four columns were locally strengthened in the potential plastic hinge region with a CFRP jacketing for investigating the effectiveness of this strengthening intervention in case of columns with borderline behavior between flexure and shear. The effect of concrete strength on the CFRP jacketing effectiveness was evaluated for two classes of concrete: two columns with poor quality concrete (*f*_c_ = 15 MPa) and two with medium quality concrete (*f*_c_ = 30 MPa) were tested. Poor quality concrete columns were strengthened with a light CFRP jacketing (i.e., one ply of CFRP) whereas medium quality concrete columns were jacketed with a strong reinforcement (i.e., two plies of CFRP). Two types of CFRP composite material (named FRPa and FRPb) were adopted, to analyse the influence of elastic stiffness and ultimate strain of the strengthening system on the specimen behavior.

To investigate the use of FRCC jacketing as an alternative to CFRP jacketing in case of columns already damaged by a seismic event or columns with deteriorated concrete cover, one poor quality concrete column was pre-damaged and then repaired and strengthened with FRCC jacketing replacing the original concrete cover.

A summary of the test matrix with the specimens’ main characteristics, such as the name, the mechanical properties and the strengthening solution, is reported in Table 1. The notation of specimens is CX_Y, where C stays for column, X refers to the concrete compressive strength (L, low or M, medium) and Y denotes the strengthening system adopted (FRPa, FRPb or FRCC). A detailed description of the strengthening solutions and the test features is reported in the following paragraphs.

### 2.2. Geometric Details and Strengthening Description

The specimens were cantilevers (i.e., single curvature), made by a column and a foundation block (1200 × 1200 × 600 mm^3^). The foundation block and the column of each specimen were cast in two different phases in order to reproduce the discontinuity of concrete at the column-foundation interface, as is common practice in concrete casting procedures.

Specimens had a square cross-section 300 × 300 mm^2^ reinforced with six 18 mm diameter deformed rebars (longitudinal geometrical reinforcement ratio, ρ_tot_ = *A*_s_/*bh* = 1.7% with *A*_s_ total area of longitudinal steel reinforcement and *b*,*h*, cross section dimensions). No additional longitudinal reinforcement, which could help in shear resisting mechanism, was provided in the column secondary direction. The concrete clear cover thickness was 20 mm on each specimen.

Transverse reinforcement was made of 8 mm diameter ties, spaced at 150 mm apart (transverse geometrical reinforcement ratio, ρ_w_ = 0.2%). A reduced spacing was adopted in the zone of load application to avoid localized damages.

**Figure 1:**
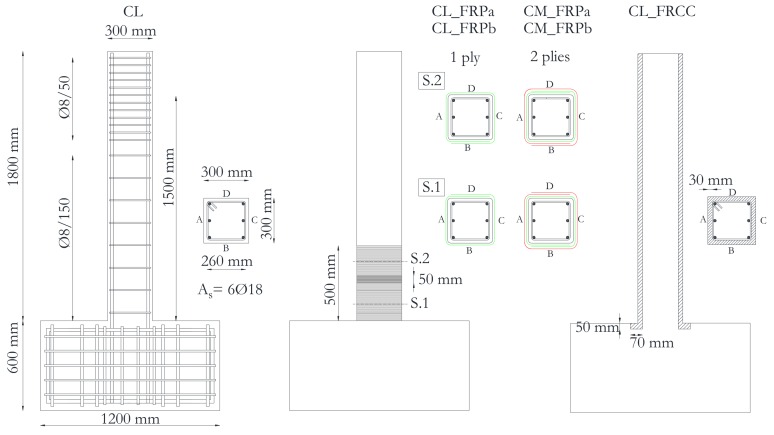
Specimens’ geometry and strengthening configurations.

The columns total height was 1800 mm, but the lateral load was applied at a distance of 1500 mm from the foundation block, in order to simulate the behavior of a typical 3000 mm height column, assuming that shear length *L_S_* is the mid-eight (i.e., *L_S_* = 1500 mm, *L_S_*/*h* = 5). The specimens’ geometry along with the strengthening intervention details are reported in Figure 1. 

**Figure 2:**
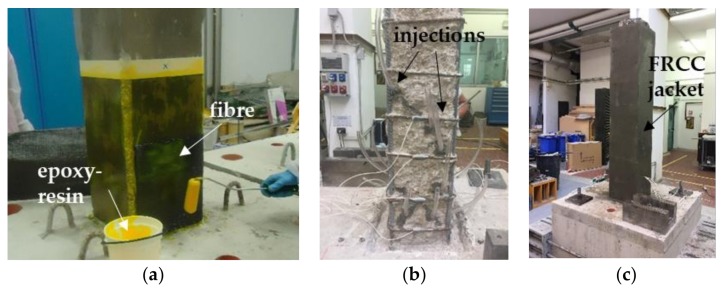
(**a**) CFRP application; (**b**) epoxy-resin injections; (**c**) FRCC jacketing.

CFRP strengthened specimens were locally jacketed at the plastic hinge region with continuous uniaxial CFRP sheets with fibres perpendicular to the column longitudinal axis, for a length of 500 mm from the foundation block. Before applying the external reinforcement, the columns corners were rounded with radius *R* = 20 mm, in order to avoid premature rupture of the fibers. Then the concrete surface was treated with the primer and the carbon sheets were applied and impregnated with epoxy-resin, as depicted in Figure 2a. The sheets overlap was more than 250 mm for each layer along the column sides orthogonal to the horizontal load action direction. Specimens characterized by low concrete strength (CL_FRPa and CL_FRPb) were reinforced with one ply of CFRP; conversely, two plies of CFRP were used for medium quality concrete specimens (CM_FRPa and CM_FRPb).

The FRCC jacketed specimen (CL_FRCC) was pre-damaged up to a residual drift ratio equal of slightly greater than 1.5%, in order to be representative of columns with poor quality concrete heavily damaged by a seismic event [25]. After the pre-damage phase, the concrete cover was completely removed and replaced with a 30 mm thick FRCC jacket, slightly enlarging the cross section of 20 mm per side. The specimen was first repaired with mortar and epoxy-resin injections (see Figure 2b) to close the flexural and shear cracks that were opened in the concrete core. The epoxy-resin injections were cured for 24 h before the FRCC jacket casting. In order to connect the jacket to the column base, a 50 mm deep and 40 mm large socket was realized in the foundation block before jacketing, see Figure 2b. By this, the jacket had an L-shape inside the foundation block, which increased the available bond surface and created a sort of anchorage between the jacket and the foundation, as depicted in Figure 1. The 30 mm thick FRCC jacket was casted inside a specific formwork, in order to fill the socket in the foundation block (Figure 2c). The jacket was cured 15 days before testing.

### 2.3. Mechanical Properties

The main mechanical properties of the materials are summarized in Table 1. Four concrete 150-mm side cubes were cast during each specimen preparation and were cured under the same environmental conditions. The mean cylindrical concrete strength, *f*_c_, derived for each specimen is reported in Table 1. The same class of steel was used for all columns: average yielding strength *f*_yl_ = 531 MPa for longitudinal reinforcement and *f*_yw_ = 525 MPa for transverse reinforcement were derived from tensile tests on coupon bars. 

As aforementioned, two types of CFRP composite material, FRPa and FRPb, with the same unit weight of 600 g/m^2^ but different ultimate strain and elastic modulus were used. The mechanical properties of the dry fiber material types declared by the manufacturers (i.e., elastic modulus of dry fibers *E*_f_ and ultimate strain *ε*_fu_) are also reported in Table 1. 

**Figure 3:**
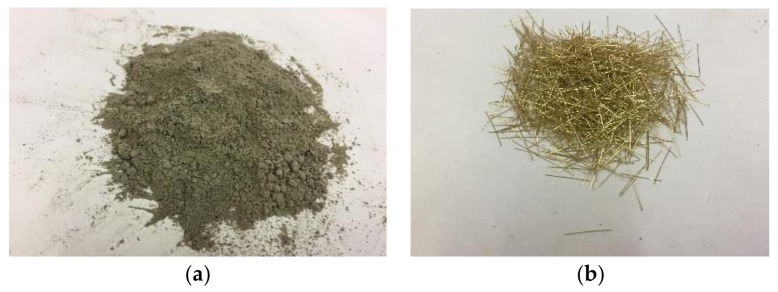
FRCC material components: (**a**) cement-based matrix; (**b**) steel fibers.

The FRCC material is characterized by a high-strength cement-based mortar with very small aggregates (maximum size 2.5 mm) and plain short steel fibres (fibre diameter 0.21 mm, fibre length 13 mm), see Figure 3. The fibers’ volumetric ratio is lower than 2%, in order to allow the workability of the composite system and to reduce the costs. This low volumetric ratio, typical of fiber-reinforced cement, provides a strain-softening behavior under tension after the opening of the first crack. 

**Figure 4:**
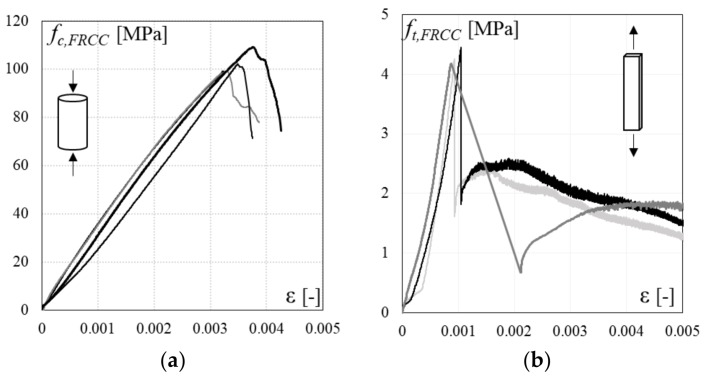
FRCC material stress-strain behavior: (**a**) compressive test; (**b**) direct tension test.

To characterize the mechanical properties of the adopted FRCC material, compression tests on cylindrical specimens (diameter 100 mm, height 200 mm) and direct tension tests on dog-bone coupon tests (width 40 mm, length 150 mm, thickness 10 mm) were performed under displacement control. The average mechanical properties derived from experimental tests on FRCC materials are summarized in Table 1: compressive strength *f*_c,FRCC_, elastic modulus *E*_c,FRCC_, ultimate compressive strain *ε*_cu_ and tensile strength *f*_t,FRCC_. Stress-strain curves for the FRCC material in both compression and tension are depicted in Figure 4a,b, respectively.

### 2.4. Test Setup, Instrumentation and Loading Protocol

**Figure 5:**
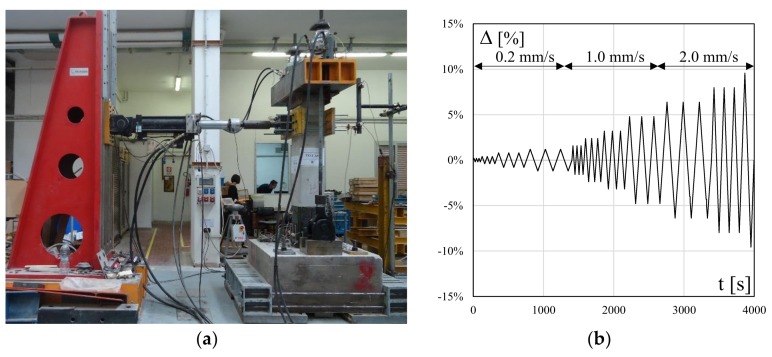
(**a**) Test setup; (**b**) loading protocol.

The full-scale columns were subjected to constant compressive axial load and horizontal cyclic displacements. The lateral displacements were applied at a distance of 1500 mm from the base cross-section by using an electrohydraulic actuator (stroke ±250 mm, maximum load +500 kN, −300 kN) integrated in a steel reaction wall and a 500 kN load cell was used to measure lateral loads. A detailed description of the test setup and relevant instrumentation is reported in [9] and shown in Figure 5a. 

All the specimens were subjected to an axial load ratio (*ν* = *N/A*_c_*f*_c_, where *N* is the compressive axial load, *A*_c_ is the concrete gross area and *f*_c_ is the mean cylindrical concrete strength) equal to 0.1 and a lateral cyclic loading under drift control (i.e., drift ratio Δ = *d*/*L_S_* with *d* equal to the horizontal applied displacement), see Figure 5b. A rate of 0.2 mm/s was adopted for the initial three cycles up to a drift ratio of 1.2%, then a higher rate of 1.0 mm/s was adopted for the next five cycles and 2.0 mm/s for the last cycles. For each cycle, the target displacement was achieved three times, see Figure 5b.

The FRCC jacket was not directly loaded by the axial load, since a gap between the jacket and the top end of the specimen was left during the casting process. This is for being more representative of realistic transmission of vertical loads during actual strengthening interventions, where the axial load is mainly carried by the original concrete core.

**Figure 6:**
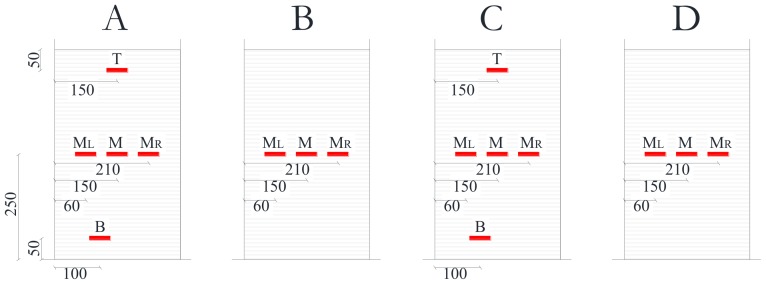
Strain gauges scheme on CFRP sheets.

Strain gauges (i.e., SGs) were placed at the mid-height and mid-span (termed M) of each CFRP jacketed column side, with the aim of monitoring axial strains in the external reinforcement. Additional SGs were installed on the flexural sides (side A and side C), at 50 mm from the top and bottom of the sheet (named T at top and B at bottom) and at corners along the mid-height (named M_L_ for left side and M_R_ for right side), to monitor the distribution of axial strains along the height of confined region and for checking strain concentration at corners. The SGs scheme for CFRP strengthened specimens is depicted in Figure 6.

**Figure 7:**
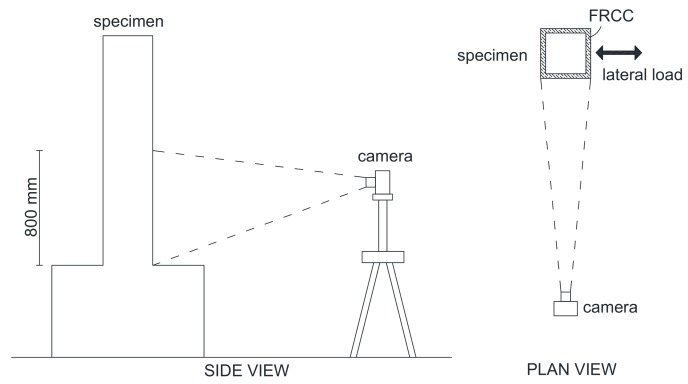
DIC system.

In the case of specimen CL_FRCC, strain gauges were put on the longitudinal (for flexural sides A and C) and transverse reinforcement (on shear sides B and D) before the FRCC jacket casting. The strain field on the shear side of the FRCC jacket was monitored by means of the Digital Imagine Correlation (DIC), see Figure 7. In particular, a region of 800 mm from the foundation block was monitored with the DIC. A high-resolution camera placed orthogonal to the shear side of the specimen took pictures of the monitored area every 6 seconds during the test. Since the shear side of the specimen remains plain during the test, the pictures taken of the specimen are not affected by any distortion. The Matlab-based code that performs 2D DIC used herein was written by Elisabeth Jones at the University of Illinois Urbana-Champaign [26]; it is a free and open-source software available on the platform Matlab’s File Exchange.

## 3. Experimental Outcomes

The main outcomes derived from the experimental program are described in the following sections in terms of the global behavior of columns and relevant failure mode as well as the effectiveness of the strengthening solutions.

### 3.1. Global Behavior

**Figure 8:**
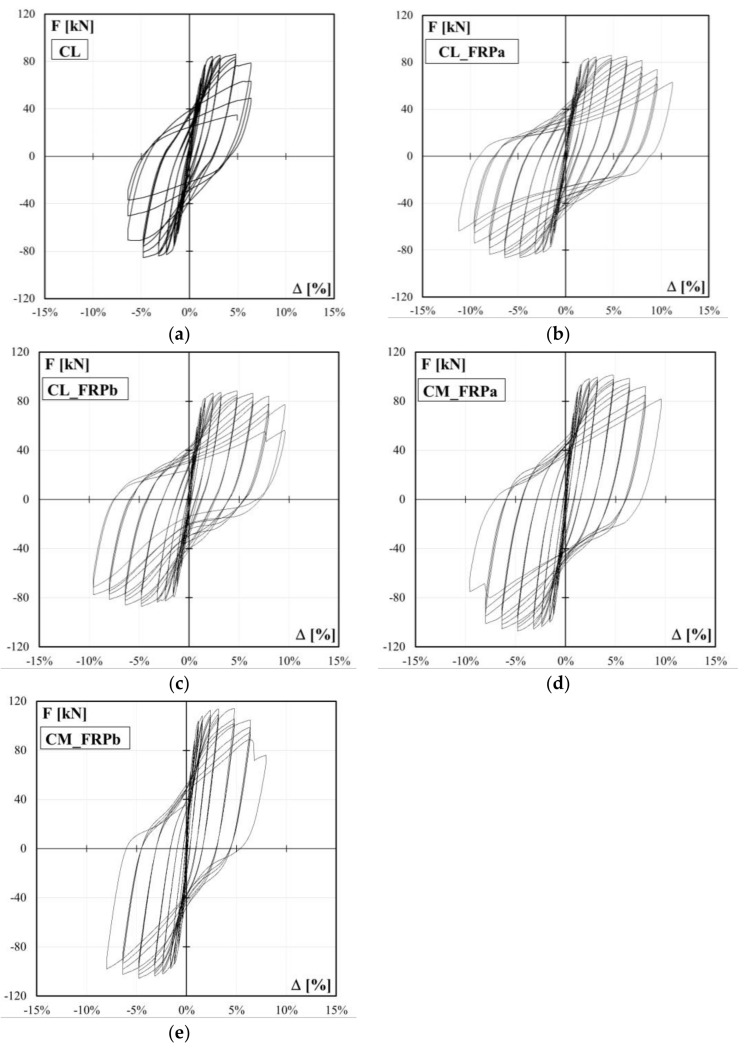
Force-drift relationships: (**a**) CL; (**b**) CL_FRPa; (**c**) CL_FRPb; (**d**) CM_FRPa; (**e**) CM_FRPb.

**Figure 9:**
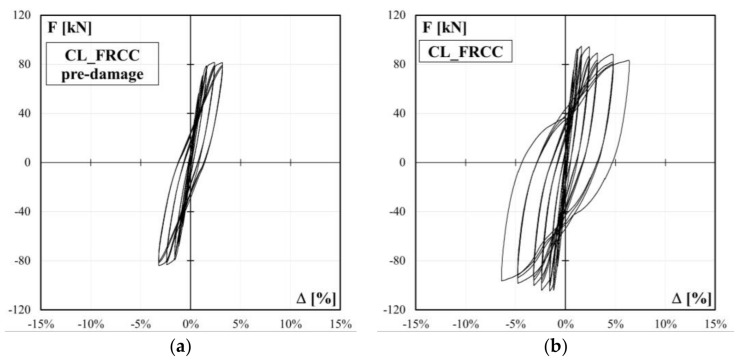
Force-drift curve for specimen CL_FRCC: (**a**) pre-damage; (**b**) after retrofitting.

The experimental lateral load-drift relationships for the control specimen, CL, and the four columns strengthened with CFRP jacketing, CL_FRPa, CL_FRPb, CM_FRPa, CM_FRPb, are depicted in Figure 8. The load-drift curves for specimen CL_FRCC experienced during the pre-damage phase and after the retrofitting are reported in Figure 9a,b, respectively. The relevant experimental outcomes for both positive and negative load-action directions for all specimens are reported in Table 2: peak forces and relative drift ratios, *F*_max_ and Δ*_F_*_max_, drift ratios at apparent yielding Δ_y_, drift ratios at conventional failure, Δ_0.8Fmax_, defined as the values corresponding to 80% of the peak force on the envelope curve, and ductility factor, *μ*_Δ_, defined as the ratio between ultimate and apparent yielding drift, and percentage of increased ductility due to the strengthening with respect to the control specimen in the positive and negative load direction, respectively, Δ*μ*_Δ_. The apparent yielding drift used for the ductility factor evaluation is assumed as the intersection point between the secant at the 70% of the peak strength and the horizontal at the peak strength on the experimental load-drift curve.

In terms of strength capacity, the CFRP jacketing of plastic hinge region has no significant influence on the peak force of columns under combined axial load and bending moment. Indeed, control column CL achieved peak strength of 86.1 (−85.4) kN and CFRP strengthened specimen, CL_FRPa and CL_FRPb, reached values of 86.0 (−86.5) kN and 88.2 (−87.2) kN, respectively. Specimens characterized by medium quality concrete, CM_FRPa and CM_FRPb, achieved similar values of flexural capacity: 101.3 (−107.1) kN and 114.1 (−105.5) kN for CM_FRPa and CM_FRPb, respectively. The FRCC jacketing produced a slight increase in flexural capacity with respect to that achieved during the pre-damage phase: the peak strength increased from 81.6 (−84.0) kN to 94.8 (−104.7) kN, as reported in Table 2.

In terms of ductility, poor quality concrete specimens CL_FRPa and CL_FRPb achieved a ductility factor of 7.2 (6.7) and 7.8 (6.5), respectively. Thus, specimen strengthened with FRPb type, characterized by higher axial rigidity, achieved a greater ductility factor with respect to specimen strengthend with FRPa type. This is due to a lower apparent yielding drift ratio experienced by CL_FRPb with respect to specimen CL_FRPa. In comparison with the control column, the CFRP local jacketing increased the ductility by 67%–60% and 81%–55% for specimens CL_FRPa and CL_FRPb, respectively. Medium quality concrete strengthened specimens achieved ductility factors comparable with those of poor quality concrete strengthened specimens: CM_FRPa and CM_FRPb reached ductility factors of 8.2 (7.5) and 6.2 (6.7), respectively. Thus, the strong CFRP strengthening combined with a medium quality concrete did not significantly improve the deformation capacity of members. Conversely, the CFRP local jacketing is more effective in the case of poor quality concrete columns.

The poor quality concrete FRCC jacketed specimen ductility factor was 6.8 (7.1). Thus, an improvement of 58%–69% with respect to control column was observed. Also in this case, this improvement in ductility factor is related to the lower apparent yielding drift ratio experienced from specimen CL_FRCC with respect to the control column.

A deep analysis of the specimens’ failure mode and of the strengthening systems behavior is discussed in the following.

### 3.2. Failure Modes and Strengthening Effectiveness

#### 3.2.1. Control Column (CL)

**Figure 10:**
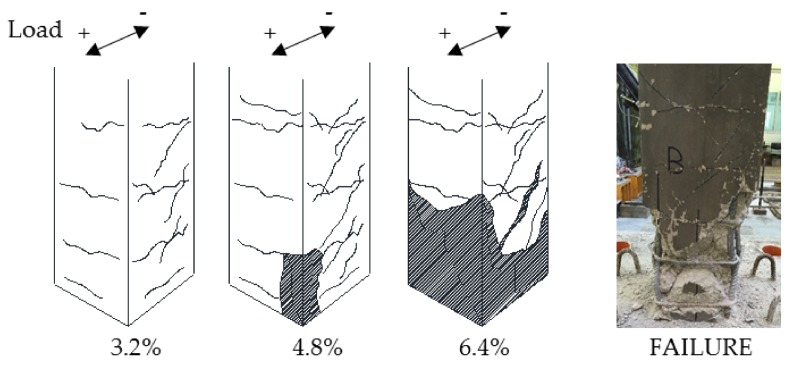
Damage evolution for control column CL.

In the control specimen, slight horizontal flexural cracks appeared at the time of the first cycles of loading in correspondence to steel transverse reinforcement. Starting from a drift ratio of 1.2%, one small diagonal cracks appeared on both shear sides of the control column. As the displacement demand increased (3.2%) the diagonal cracks expanded and extended on the shear sides of the specimen. At a drift ratio of 4.8%, the buckling of a corner longitudinal bar caused the spalling of the concrete cover. After the imposed drift ratio of 6.4%, the concrete in the plastic hinge region was highly deteriorated and a large shear crack crossing the core was opened. The control column damage evolution is depicted in Figure 10, along with a picture of the specimen at failure.

Thus, the specimen exhibited a flexural behavior up to the imposed drift ratio of 6.4%, when a sudden drop of lateral capacity and stiffness caused by the shear interaction in the plastic hinge region was observed (see Figure 8a) and the test ended for safety reasons. The degradation of concrete in the plastic hinge region, the quite large spacing of transverse reinforcement and the absence of longitudinal reinforcement in the secondary direction probably contributed to the borderline behavior of the column between flexure and shear. 

**Figure 11:**
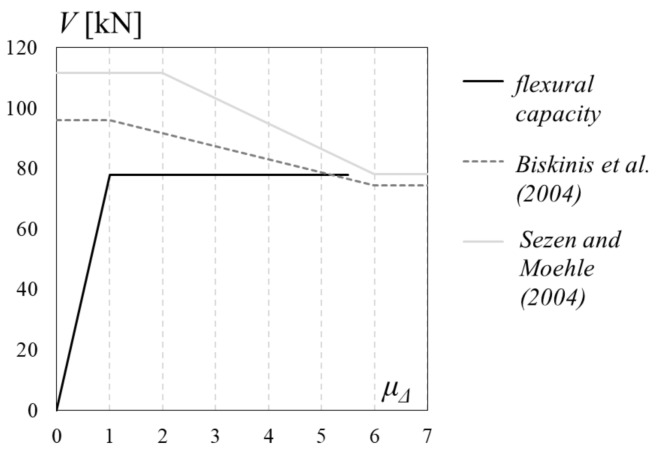
Theoretical capacity assessment.

The control specimen confirmed the expected failure mode of the column that was influenced by the shear interaction mechanism. Theoretical predictions for the shear capacity of the control column, according to degrading capacity models proposed by Biskinis et al. 2004 [13] and Sezen and Moehle 2004 [14], were compared with the specimen’s theoretical flexural capacity, and evaluated from cross-sectional analysis, in Figure 11. Corrective factors suggested in [15] have been adopted for improving the accuracy of adopted shear capacity models. Theoretical yielding and ultimate drift ratios were calculated following Eurocode 8—Part 3 [27] provisions. The comparison between flexural and shear capacity showed that the shear capacity reduced by the ductility demand is very close to the flexural capacity expected for the specimen. However, it should be noted that shear capacity models are not able to predict with a sufficient accuracy the drift at which the shear interaction will happen [28], due to the error related to the slope of degrading brunch in the capacity curve. The experimental outcomes for the control specimen attested that the shear interaction happened before the flexural failure, at a ductility factor of 4.3, as already reported in Table 2 and slightly before theoretical prediction depicted in Figure 11.

#### 3.2.2. CFRP Strengthened Columns (CL_FRPa, CL_FRPb, CM_FRPa, CM_FRPb)

The CFRP local jacketing of the potential plastic hinge region avoided the sudden drop of lateral capacity observed in the control specimen and, consequently, the shear interaction mechanism, as observed from the comparison of load-drift capacity curves in Figure 8.

**Figure 12:**
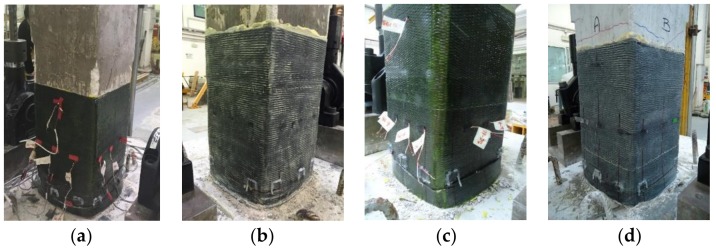
Damage at failure: (**a**) CL_FRPa; (**b**) CL_FRPb; (**c**) CM_FRPa; (**d**) CM_FRPb.

In particular, poor quality concrete specimen CL_FRPa achieved the conventional flexural failure caused by the strength degradation. No FRP tensile rupture or debonding phenomena were recognized on the external CFRP jacketing. However, longitudinal cracks between fibers, caused by the high lateral pressure exerted by the deteriorated concrete inside the jacket, were observed at a distance of 50 mm from the base (see Figure 12a).

Specimen CL_FRPb, also characterized by poor quality concrete but strengthened with a composite material with higher axial rigidity with respect to CL_FRPa, experienced a flexural behavior up to a drift ratio of 9.6%, when the tensile rupture of one longitudinal rebar caused a drop of the lateral capacity and the test was arrested. Thus, in this case, the conventional failure was not achieved. Longitudinal crack between fibers was observed on the jacket, but in a lower ratio with respect to specimen CL_FRPa (see Figure 12b). The reduced crack opening in the jacket produced a slower post-peak strength degradation for specimen CL_FRPb in comparison with CL_FRPa (Figure 8b,c).

The medium quality concrete specimen CM_FRPa experienced the flexural conventional failure in the positive load action direction, whereas in the negative one the tensile rupture of a longitudinal rebar was achieved for a drift ratio of −9.6% (see Figure 8d). The composite system worked similarly to that of specimen CL_FRPa, without any debonding or local tensile rupture but with evident longitudinal cracks at a distance of 50 mm from the base section, as visible in Figure 12c.

Conversely, specimen CM_FRPb did not achieve the conventional failure due to a local rupture of a longitudinal steel bar during the second repetition of the last cycle, which caused a degradation of lateral strength, and the test was arrested. A few small diagonal cracks were detected on the shear sides in the unconfined region, but no significant strength degradation due to shear was recorded. In this case, no longitudinal cracks between fibers were observed, see Figure 12d. However, large cracks at the interface between the column and the foundation block were observed, more than other specimens, indicating that a consistent component of the drift ratio was represented by the base rotation.

To compare the effectiveness of the strengthening systems for different concrete strength and CFRP axial rigidities, the effective strains on the CFRP jacket due to combined shear action and concrete lateral dilatation were investigated. The CFRP strains were measured by means of strain gauges located at mid-span and mid-height of each side of the specimens. Additional strain gauges were also installed at the top and bottom and near corners on flexural sides.

**Figure 13:**
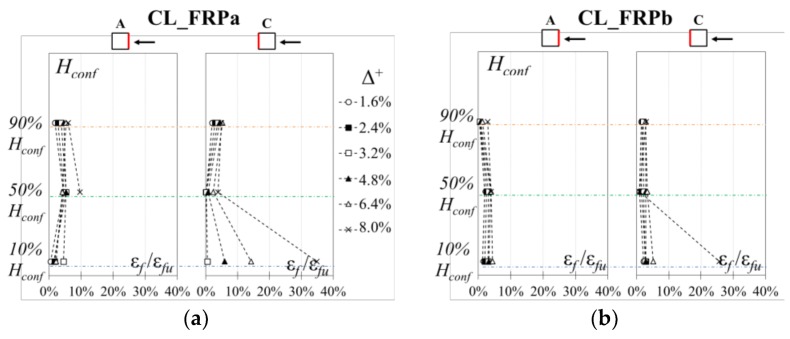

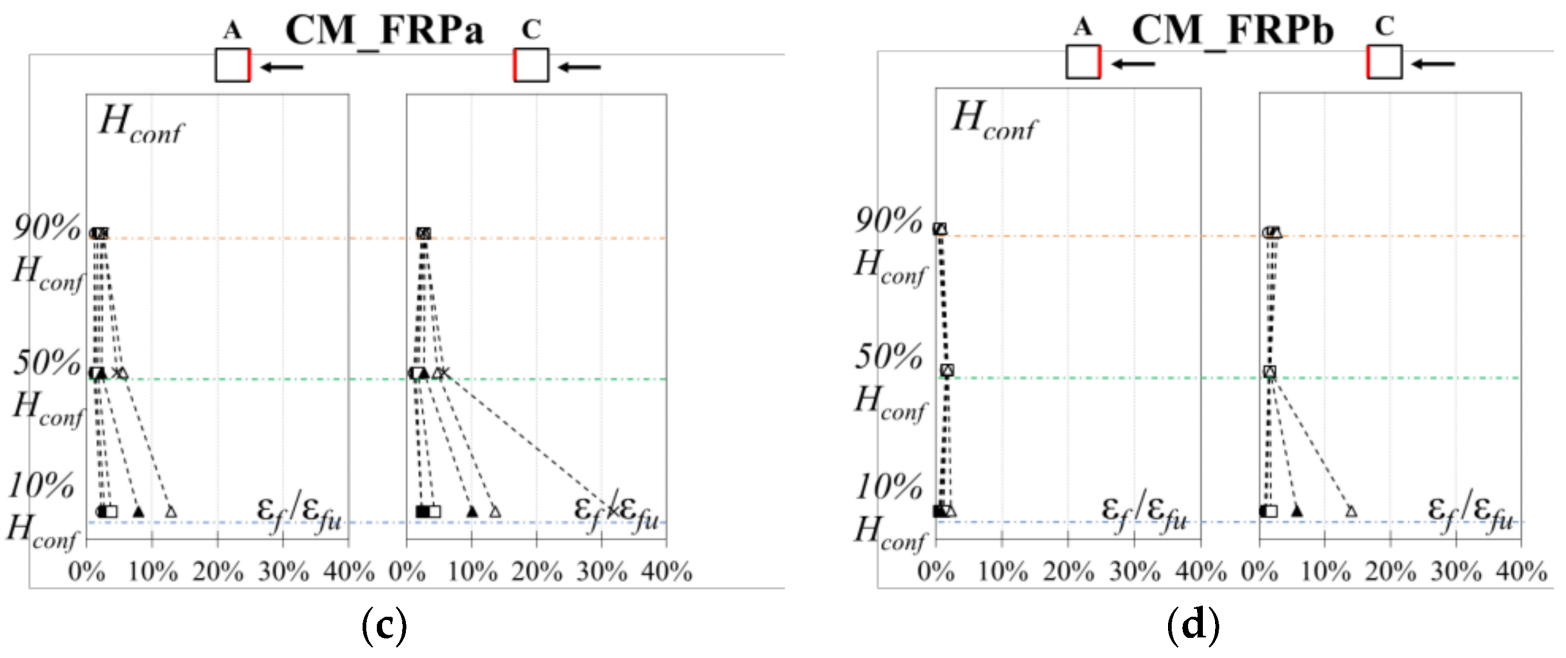
Vertical CFRP axial strain profiles: (**a**) CL_FRPa; (**b**) CL_FRPb; (**c**) CM_FRPa; (**d**) CM_FRPb.

Firstly, the vertical strain profiles on CFRP sheets were analyzed, to monitor the effectiveness of the strengthening system along the height of the jacketed region. In particular, the ratios between CFRP experimental strains recorded at positive peak drift ratios of each cycle, *ε*_f_, and ultimate composite material strain, *ε*_fu_, are depicted in Figure 13 for different positive imposed drift ratios up to failure along the CFRP sheet height, *H*_conf_ (i.e., 10% *H*_conf_, 50% *H*_conf_ and 90% *H*_conf_). It should be noted that carbon fibers in the plastic hinge region work simultaneously for shear action and for confinement, contrasting the concrete lateral dilatation caused by a non-uniform distribution of compressive stresses given by the combined action of axial load and bending moment. Thus, in the positive load action direction, column side C is subjected to compression whereas side A is in compression for very small imposed drift ratios, otherwise it is in tension.

The carbon fibers effective strains recorded on CFRP sheets are linearly distributed along the confined region height. Strain concentrations are observed at the bottom of all specimens, where the longitudinal cracks between fibers were developed. These stress/strain concentrations are mainly caused by the high damage of concrete inside the CFRP jacket, which is disaggregated and exerts a high lateral pressure at the column base. Peak strains ratios of 34.9%*ε*_fu_ and 25.6%*ε*_fu_ were recorded at the bottom of specimens CL_FRPa and CL_FRPb, strengthened with one ply of CFRP. Conversely, peak strain of 31.9%*ε*_fu_ and 14.0%*ε*_fu_ were recorded for specimens CM_FRPa and CM_FRPb, strengthened with two plies of CFRP, respectively.

**Figure 14:**
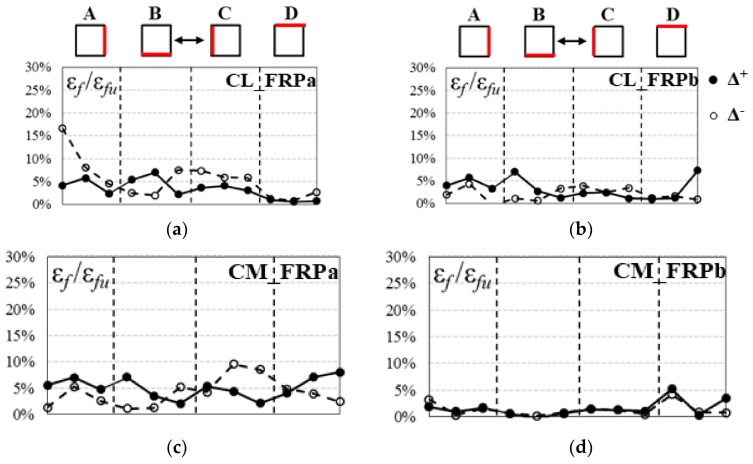
Horizontal CFRP axial strain profiles at a drift ratio of 6.4%: (**a**) CL_FRPa; (**b**) CL_FRPb; (**c**) CM_FRPa; (**d**) CM_FRPb.

The distribution of fibers axial strains along the perimeter of the specimens was also investigated. The horizontal strain ratio profiles recorded at the mid-height of each columns side are reported in Figure 14 for all specimens at a drift ratio of 6.4%, before the rupture of longitudinal bars. For each side of the specimen, the strain at mid-span and at corners were monitored. 

The horizontal strain ratios were not perfectly symmetric for the positive and negative load action directions for almost all the CFRP strengthened specimens. The axial strains were not uniformly distributed along the columns perimeter of all specimens, as visible in Figure 14. However, a clear trend of horizontal profiles was not clearly visible and the influence of the strain gradient caused by the bending moment did not influence significantly the distribution of strains along the perimeter. The peak strains recorded on shear sides B and D were probably caused by the development of cracks crossing the monitored point. In all specimens, axial strains recorded near corners were generally lower than those recorded at the mid-span of the jacket, probably due to geometrical reasons that avoid the full transmission of strains near corners. 

The axial strains ratios recorded at the mid-height were usually smaller than 10%*ε*_fu_, expect for a peak of 16.6% *ε*_fu_ recorded on the corner of side A for specimen CL_FRPa.

It should be also observed that strain ratios recorded on specimen CM_FRPb (Figure 13d) are particularly low if compared with other specimens. This implies that the composite system did not work in the post-peak phase, probably because the base rotation was prevalent with respect to flexural deformations and the specimen actually behaved as a rigid body under rocking.

No significant differences in terms of fibers effective strains can be observed among specimens with different concrete compressive strength or composite axial rigidity.

#### 3.2.3. FRCC Strengthened Column (CL_FRCC)

As aforementioned, the specimen was first pre-damaged, according to the load protocol depicted in Figure 5b, up to a drift ratio of 3.2%. At the end of the pre-damage phase, the residual drift was 1.6%, see the force-drift relation in Figure 9a.

**Figure 15:**
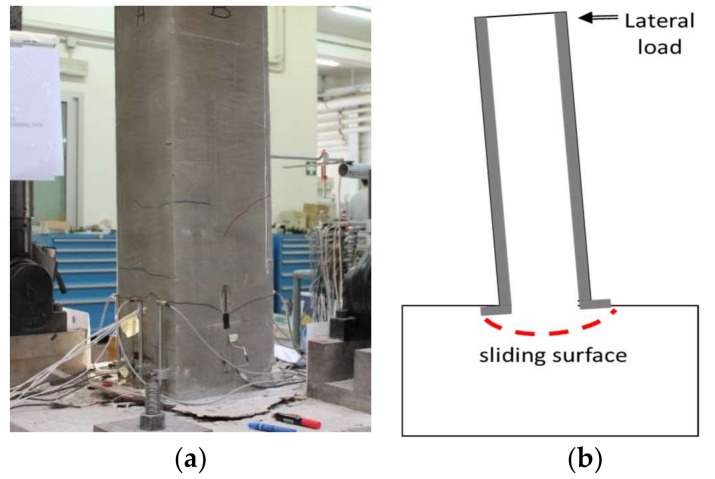
(**a**) FRCC jacket at the end of the test; (**b**) failure mode.

The repaired and strengthened specimen exhibited a flexural behavior up to a drift ratio of 6.4%, see Figure 9b. After this drift ratio, the test was arrested for safety reasons and the defined conventional failure was not achieved. At the end of the test, only a few small cracks were detected on the jacket (see Figure 15a) and no specific failure mechanisms, such as shear cracks or longitudinal bars rupture, were observed. This is due to the anchorage system adopted for the FRCC jacket, which induced a rigid rocking behavior of the column along a sliding surface inside the foundation block, as visible from Figure 15b. Thus, the FRCC jacket avoided the sudden failure observed in the control specimen but, without a proper anchorage, shifted the probable future failure mechanism at the interface between jacket and foundation. 

**Figure 16:**
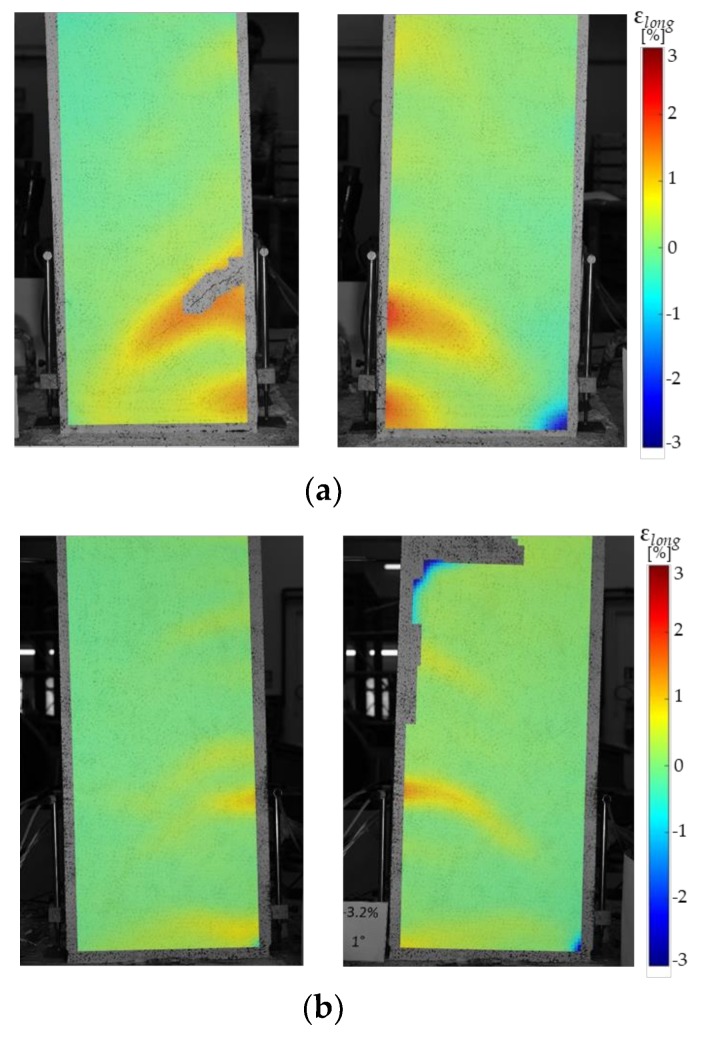
Strain fields for specimen CL_FRCC during pre-damage (**a**) and repaired phase (**b**).

The longitudinal strain fields derived from the digital imagine correlation (DIC) for specimen CL_FRCC before and after the jacketing at a drift ratio of 3.2% in both positive and negative load action directions are depicted in Figure 16. In bare column, the tensile strains concentration in correspondence to a diagonal crack opened in the plastic hinge region is clearly visible in both load action directions (Figure 16a,b). The diagonal crack started opening from a horizontal crack developed on the flexural side in correspondence to the second stirrup. Furthermore, the trend of tensile strains distribution over the column length was in accordance with the truss analogy model. After the retrofitting, the stain distribution over the FRCC jacket confirmed the rocking behavior of the specimen. Indeed, very low longitudinal strains were recorded on the jacket and only small strains concentrations were observed in correspondence to the second stirrup, but no cracks on the jacket were detected.

## 4. Discussion and Comparison between Strengthening Techniques

**Figure 17:**
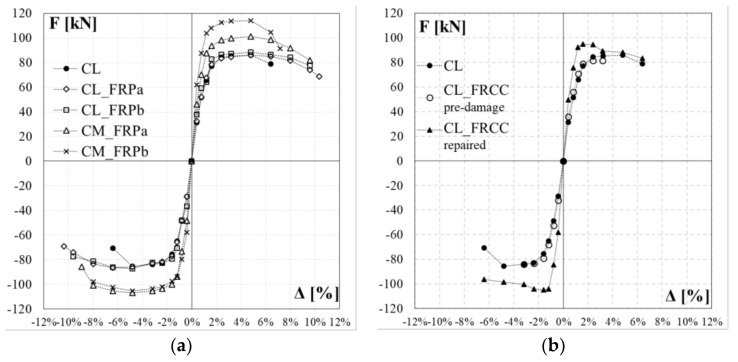
Envelope curves: comparison between control specimen and (**a**) CFRP strengthened columns; (**b**) pre-damaged and FRCC strengthened columns.

To better compare the performance of CFRP or FRCC jacketed specimens with respect to the control specimen, the envelope curves for both positive and negative drift ratios are reported in Figure 17. In particular, the envelope curves of the control column and CFRP strengthened specimens are depicted in Figure 17a. Conversely, those of the control column and of CL_FRCC during the pre-damage phase and after the retrofitting are depicted in Figure 17b.

From the comparison of the envelope curves in Figure 17a, specimen CL_FRPa exhibited a slightly faster strength degradation in the post-peak phase with respect to CL_FRPb.

Both specimens, characterized by medium quality concrete and strengthened with two plies of CFRP, had a larger initial lateral stiffness if compared with all the poor quality concrete specimens. Specimen CM_FRPa exhibited a performance very similar to poor quality concrete strengthened specimens but with a faster strength degradation in the post-peak phase. Conversely, specimen CM_FRPb had a poor performance if compared with companion specimen CM_FRPa, with a strong post-peak strength degradation in the positive direction caused by the premature rupture of the longitudinal bar.

Specimen CL_FRCC exhibited a very similar response of control column during the pre-damage phase (Figure 17b). After the FRCC jacketing, an increase in terms of lateral stiffness and strength was achieved. A sudden strength degradation was observed at a drift ratio of 2.4%, when probably started the rocking behavior. After this drift ratio, the contribution of the FRCC jacket was almost negligible and the following branch of the envelope curve followed the envelope of control specimen.

In the following, the cyclic performance of specimen retrofitted with FRCC jacketing is compared with those of CFRP strengthened specimens in terms of stiffness degradation, energy dissipation and damage index. This clearly show that the system was able to repair the column but the effectiveness in terms of strengthening was limited due to the attainment of a premature failure mode at the column base.

### 4.1. Stiffness Degradation

**Figure 18:**
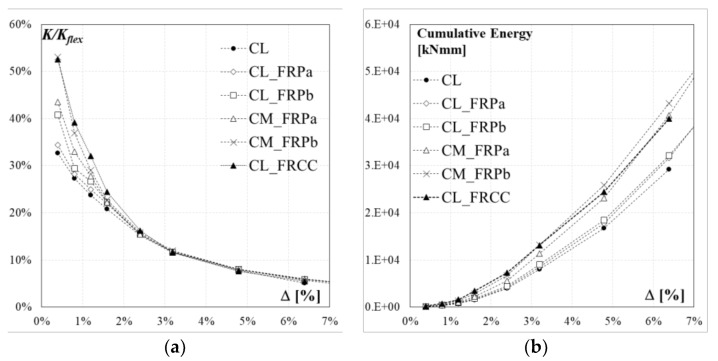
(**a**) Secant stiffness degradation; (**b**) cumulative energy dissipation.

To investigate the effects of the strengthening techniques on the columns stiffness degradation caused by the damaging under cyclic loading, the experimental stiffness was computed as the peak-to-peak secant stiffness of the first cycle of each imposed drift ratio. The ratio between the experimental secant stiffness, *K*, and the theoretical elastic stiffness, *K*_flex_ = 3*EI*_g_/*L_S_*^3^ (where *E* is the concrete elastic modulus and *I*_g_ is the gross section inertia), is plotted for the six RC columns as a function of the drift ratio in Figure 18a. The ratios *K*/*K*_flex_ for tested specimens are also summarized in Table 3.

At first cycle, the ratio *K*/*K*_flex_ is 33% in the case of the control column, 34% for specimen CL_FRPa and 41% for specimen CL_FRPb. Specimens with medium quality concrete, CM_FRPa and CM_FRPb, achieved initial ratios of 43% and 53%, respectively. However, after a drift ratio of 1.6%, the stiffness degradation followed the same parabolic trend up to failure for all specimens.

The FRCC jacketed specimen achieved a greater lateral stiffness (53%) with respect to the control specimen for first cycles up to a drift ratio of 2.4%. Compared with CFRP strengthened specimens, specimen FL_FRCC experienced a very similar stiffness degradation as specimen CM_FRPb, characterized by a medium quality concrete and strengthened with two plies of CFRP.

### 4.2. Energy Dissipation

The cumulative dissipation energy due to the cyclic loading, evaluated at the end of each cycle as the area below the force-drift hysteretic curve, is reported as a function of the drift ratio for the six specimens in Figure 18b. The results of the cumulative energy are also reported in Table 4.

A slight difference in the cumulative dissipation energy trend is observed for the control specimen, CL, and the specimens strengthened with one ply of CFRP, CL_FRPa and CL_FRPb. Conversely, specimens CM_FRPa and CM_FRPb, characterized by medium quality concrete and strengthened with two plies of composite system, dissipated more energy with respect to other specimens, since from the first cycles. Thus, the combination of higher confinement effect and better quality concrete reduced the pinching phenomenon. 

The FRCC jacketed specimen CL_FRCC dissipated more energy with respect to the undamaged control specimen and the other two poor quality concrete strengthened specimens, since the first cycles. This is mainly related to the reduced pinching effect observed in the force-drift relation in Figure 9b. The dissipative capacity of specimen CL_FRCC is very close to that of specimen CM_FRPa and CM_FRPb. 

### 4.3. Damage Index

The damage evolution is investigated by means of a cumulative damage index, which give a numerical indication of the damage for the RC member. This cumulative index is obtained by cumulating the damage achieved during each loading cycle.

Recent research showed that the RC members damage is dependent on the residual deformation and on the energy dissipated at the end of each cycle [29,30,31,32]. The combined damage index proposed by Park and Ang [30], reported in Equation (1), can be a suitable parameter for evaluating the damage of RC members during cyclic actions:

(1) $$DI=\frac{d}{d_{u}}+\frac{\beta }{d_{u}F_{y}}\int dE$$

where *d* is the peak displacement achieved at each cycle, *d*_u_ is the specimen ultimate displacement, *F*_y_ is the yielding strength and *dE* is the increment in absorbed hysteretic energy. The constant parameter *β* gives the ratio of the incremental damage caused by an increase of the maximum response to the normalized incremental hysteretic energy and it is taken equal to 0.25 for slightly reinforced structures [32].

**Figure 19:**
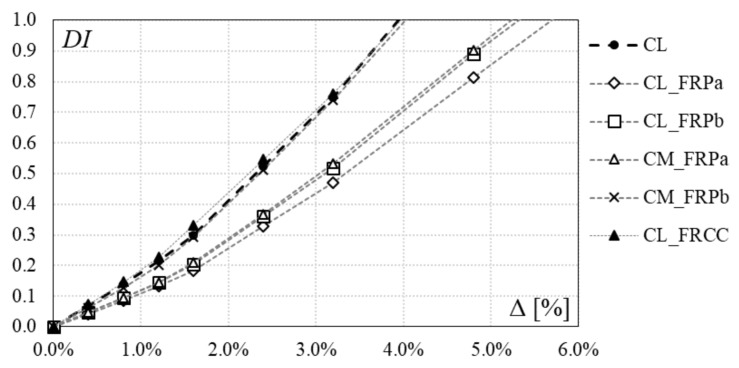
Damage index.

The damage index, DI, calculated at each cycle for all specimens is reported in Table 5 and depicted in Figure 19. Greater values of DI have been achieved from the control specimen CL, with respect to CFRP strengthened specimens CL_FRPa, CL_FRPb and CM_FRPa. Specimen CM_FRPb, that experienced a poor performance despite the CFRP strengthening, achieved the same damage index as the control specimen. Jacketed specimen CL_FRCC experienced the same damage evolution of the control specimen, even though the initial pre-damage phase caused a significant residual drift. Thus, the repair and jacketing intervention allowed the restoration of the original behavior.

## 5. Conclusions

Six full scale RC columns, representative of existing members with a borderline behavior between flexure and shear, were tested under constant axial load and cyclic lateral displacements. Four specimens were jacketed in the potential plastic hinge region with CFRP sheets and the influence of concrete quality (poor and medium) and composite system axial rigidity were investigated. The use of a FRCC jacketing as a valid alternative to FRP strengthening has been also investigated for poor quality concrete columns. The comparison between strengthening techniques has been developed.

Based on experimental evidence, the following conclusions can be drawn:

1. The control specimen, non-conforming to current standards, was not able to fully develop its ductility capacity, due to the buckling of longitudinal bars and the shear interaction mechanism, as typically observed in damaged structures after recent earthquakes.
2. Specimens jacketed with CFRP in the potential plastic hinge region experienced a flexural behavior avoiding the shear interaction mechanism, with significant ductility improvement with respect to the control specimen.
3. The CFRP jacketing were more effective in the case of poor quality concrete columns. The axial rigidity of composite system influenced the columns initial lateral stiffness and the post-peak strength degradation.
4. FRCC jacketing can be seen as a viable alternative to FRP external reinforcement, since the jacket avoided failure phenomena like bars bucking and shear interaction mechanism and significantly reduced the concrete deterioration. Furthermore, an increase in lateral capacity and energy dissipation was observed.
5. More research is needed to avoid the rocking behavior, by providing a proper anchorage with the foundation able to better increase the flexural capacity and the ductility.

## Figures and Tables

**Table 1:** Test matrix.

Test	Concrete	Steel		CFRP			FRCC				Strengthening Thickness
	*f*_c_	*f*_y,long_	*f*_y,tran_	n° Layers	*E*_f_	*ε*_fu_	*f*_c,FRCC_	*E*_c,FRCC_	*ε*_cu_	*f*_t,FRCC_	*t*_f_
	[MPa]	[MPa]	[MPa]		[GPa]	[%]	[MPa]	[GPa]	[%]	[MPa]	[mm]
CL	16.3	525	531	-	-	-	-	-	-	-	-
CL_FRPa	14.9			1	230	1.3	-	-	-	-	0.33
CL_FRPb	16.0			1	252	1.9	-	-	-	-	0.33
CM_FRPa	29.1			2	230	1.3	-	-	-	-	0.66
CM_FRPb	33.3			2	252	1.9	-	-	-	-	0.66
CL_FRCC ^1^	15.1			-	-	-	104.3	31.3	4.0	4.3	30.00

^1^ Pre-damaged specimen.

**Table 2:** Experimental outcomes.

Test		*f*_c_	*F*_max_	Δ_y_	Δ_Fmax_	Δ_0.8Fmax_	*μ*_Δ_	Δ*μ*_Δ_
		[MPa]	[kN]	[%]	[%]	[%]	[–]	[%]
CL		16.3	86.1	1.5	4.8	6.4 ^1^	4.3	-
			−85.4	−1.5	−4.8	−6.4 ^1^	4.2	-
CL_FRPa		14.9	86.0	1.4	4.8	10.4	7.2	+67%
			−86.5	−1.5	−4.8	−10.4	6.7	+60%
CL_FRPb		16.0	88.2	1.2	4.8	9.6 ^1^	7.8	+81%
			−87.2	−1.5	−4.8	−9.6 ^1^	6.5	+55%
CM_FRPa		29.1	101.3	1.2	4.8	9.6	8.2	-
			−107.1	−1.2	−4.8	−8.9	7.5	-
CM_FRPb		33.3	114.1	1.2	4.8	7.2	6.2	-
			−105.5	−1.2	−4.8	−8.0	6.7	-
CL_FRCC	pre-damage	15.1	81.6	1.5	2.4	-	-	-
			−84.0	−1.5	−3.2	-	-	-
	repaired	15.1	94.8	0.9	1.6	6.4 ^1^	6.8	+58%
			−104.7	−0.9	−1.6	−6.4 ^1^	7.1	+69%

^1^ Last drift recorded not corresponding to the conventional failure.

**Table 3:** Stiffness degradation.

Drift			*K*/*K*_flex_				
[%]	CL	CL_FRPa	CL_FRPb	CM_FRPa	CM_FRPb	CL_FRCC	
						Pre-Damage	Repaired
0.4	0.33	0.34	0.41	0.43	0.53	0.38	0.53
0.8	0.27	0.28	0.29	0.33	0.37	0.30	0.39
1.2	0.24	0.25	0.27	0.28	0.29	0.26	0.32
1.6	0.21	0.22	0.22	0.22	0.23	0.22	0.24
2.4	0.15	0.15	0.15	0.15	0.16	0.15	0.16
3.2	0.12	0.12	0.12	0.12	0.12	0.12	0.12
4.8	0.08	0.08	0.08	0.08	0.08		0.08
6.4	0.05	0.06	0.06	0.06	0.06		0.05
8.0	0.33	0.05	0.05	0.04	0.04		
9.6		0.03	0.04	0.03			

**Table 4:** Cumulative energy dissipation.

Drift			Cumulative Energy [kNmm]				
[%]	CL	CL_FRPa	CL_FRPb	CM_FRPa	CM_FRPb	CL_FRCC	
						Pre-Damage	Repaired
0.4	94.6	100.4	117.1	130.8	155.4	110.4	226.9
0.8	338.6	361.5	402.7	435.1	520.6	385.1	729.5
1.2	788.1	815.7	885.2	991.3	1292.4	854.0	1616.5
1.6	1560.4	1594.6	1801.7	2235.4	2844.6	1789.0	3422.9
2.4	3985.4	4189.7	4515.6	5640.4	6731.8	4507.7	7277.3
3.2	8022.7	8494.7	9015.9	11,418.5	13,174.4	8829.0	13,147.6
4.8	16,703.8	17,713.7	18,498.5	23,180.9	25,828.8		24,482.4
6.4	29,211.5	31,677.9	32,174.9	40,650.5	43,175.0		40,012.2
8.0		49,145.8	48,041.3	61,540.5	60,991.3		
9.6		67,001.0	64,626.2	82,146.2			

**Table 5:** Damage Index.

[%]	CL	CL_FRPa	CL_FRPb	CM_FRPa	CM_FRPb	CL_FRCC
						Repaired
0.4	0.1	0.0	0.0	0.0	0.1	0.1
0.8	0.1	0.1	0.1	0.1	0.1	0.1
1.2	0.2	0.1	0.1	0.1	0.2	0.2
1.6	0.3	0.2	0.2	0.2	0.3	0.3
2.4	0.5	0.3	0.4	0.4	0.5	0.5
3.2	0.7	0.5	0.5	0.5	0.7	0.8
4.8		0.8	0.9	0.9		
